# Self-Reported Oral Health Knowledge and Practices During Pregnancy and Their Social Determinants in Poland

**DOI:** 10.3290/j.ohpd.a44442

**Published:** 2020-07-04

**Authors:** Angelika Kobylińska, Nicole Sochacki-Wójcicka, Dariusz Gozdowski, Dorota Olczak-Kowalczyk

**Affiliations:** ^a^ Research and Teaching Assistant, Department of Pediatric Dentistry, Medical University of Warsaw, Poland. Study concept, data collection, preparation of the first draft of the manuscript, read and approved the final version of the manuscript.; ^b^ PhD Student/Researcher, 1st Clinic and Department of Obstetrics and Gynecology, Medical University of Warsaw, Poland. Study concept, data collection.; ^c^ Assistant Professor, Department of Experimental Design and Bioinformatics, Faculty of Agriculture and Biology, Warsaw University of Life Sciences, Poland. Performed statistical analysis, proofread the manuscript.; ^d^ Professor, Head of the Department of Pediatric Dentistry, Medical University of Warsaw, Poland. Study concept, preparation of the first draft of the manuscript, read and approved the final version of the manuscript.

**Keywords:** health behaviours, sociodemographic factors, dental visit, gynaecologist

## Abstract

**Purpose:**

To determine the effects of sociodemographic and pregnancy-related factors on oral health attitudes during pregnancy, as well as the main predictors of proper oral practices.

**Materials and Methods:**

An electronic survey consisting of 47 (single or multiple-choice) questions was conducted in women up to 3 years after childbirth in 2017. Sociodemographic data, as well as information on the course of pregnancy and delivery, oral knowledge and basic behaviours during pregnancy were collected. The Chi-square test and Spearman’s rank correlation coefficient were used for statistical analysis. Odds ratios were determined. A statistical significance level of 0.05 was used.

**Results:**

A total of 2480 questionnaires completed by women aged between 13 and 45 years who were up to 3 years after delivery, were analysed. Correct answers to all questions regarding basic oral health and oral practices were obtained by 20.8% and 19.6% of respondents, respectively. Proper health behaviours were more strongly correlated with the level of knowledge (r = 0.155; odds ratio (OR) = 2.44; CI:1.93–3.07; p <0.001) and the use of dental care before pregnancy (r = 0.187; OR = 2.88; CI:2.29–3.63; p <0.001) rather than age (r = 0.144), good or very good financial status (r = 0.110), high level of education (r = 0.081), urban residence (r = 0.058) or occupational activity (r = 0.049). Attending dental visits depended on the conviction about their safety (r = 0.195; OR = 2.47; CI: 2.09–2.93; p <0.001) as well as gynaecologist’s referral in the case of general conditions in pregnancy (r = 0.052) and the risk of premature birth (r = 0.053). No effects of other parameters associated with pregnancy or delivery were confirmed.

**Conclusion:**

Health attitudes during pregnancy are modified by sociodemographic factors. The main predictors of proper health behaviours include high level of knowledge on oral health and the use of dental care before conception. Furthermore, dental attendance among pregnant women depends on the awareness of the safety of dental visits and a gynaecologist referral in case of general condition and risk of premature birth.

Deteriorated oral health in pregnant women may affect the course of pregnancy, as well as the general health of the mother and her child. Oral infections in pregnancy, periodontitis in particular, increase the risk of premature birth and low birth weight.^16^,^17,25,37^ A high level of cariogenic bacteria as a result of untreated maternal tooth decay is a risk factor for early childhood caries.^5,6,11,19,36,46^ Therefore, enhancement of preventive and therapeutic interventions in the area of oral health is necessary during pregnancy.^1,2,10,13,25,38,40^ Different strategies are implemented to improve pregnant women’s oral health.^1,2,10,13,21,28,38,40,49^ In Poland, pregnant and puerperal women are entitled to free dental procedures, which enable regular check-ups, preventive and therapeutic procedures, as well as individual health education. In 2011, the Ordinance of the Minister of Health resulted in the implementation of standards for the management in pregnancy and puerperium, which include promoting a healthy lifestyle (including oral health) by midwives, oral check-ups performed by the attending gynaecologist–obstetrician and a dental check-up at 9–10 weeks of pregnancy. Group education in the field of oral health was also attempted (eg, by childcare schools). However, the rates of inadequate oral health behaviours during pregnancy in Poland (ie, low dental attendance, indicate insufficient effectiveness of these solutions).^9,32^ In other countries offering free dental care during pregnancy, pregnant women are not necessarily aware of this.^18,27,34^ This indicates the need to modify the approach to oral health promotion and enhancement among women showing improper health attitudes.

Gynaecologists and midwives are in a strategic position providing the oral health-related information and also referral service to pregnant women.^1,2,10,13,21,25,28,38,40,49^ They are well informed about perinatal oral health and are supportive of dental procedures, but unfortunately they seldom focus on oral healthcare during their prenatal care.^23^ There are many barriers for appropriate oral healthcare of pregnant patients. The most common include the sense of lack of competence, training and time.^39,48^ However, not all women require oral health education. The levels of their knowledge and oral practices also vary.^3,4,^^7,9,12,15,22,27,29,32,35,40,42,47,48^ The decision to implement education, both individual and collective, as well as its level should be based on the identified needs of women. Therefore, it is important in clinical practice to know simple indicators allowing for rapid determination of health attitudes in a given woman. The aim of the study was to identify and determine the effects of sociodemographic and pregnancy-related factors on oral health attitudes during pregnancy as well as the main predictors of proper oral practices.

## MATERIALS AND METHODS

The anonymous, electronic survey (47 single or multiple-choice questions) addressed Polish women with a history of childbirth within 3 years from the date of completing the questionnaire. The link to the questionnaire was posted on the blog website ‘Mama ginekolog’ (‘a gynaecologist mum’) in April and May 2017. The questionnaire inquired about sociodemographic data (age on the day of pregnancy termination and current age, place of residence, education, financial status, occupational activity), pregnancy and delivery (comorbidities, delivery date, pregnancy termination), health behaviour and health awareness (the frequency of toothbrushing, the use of toothpaste with fluoride, smoking, safety and the need to use dental care during pregnancy, utilisation of dental care in pregnancy and the relationship between the oral health of the mother and her child). Questions in the questionnaire were assessed by the Bioethical Committee of the Medical University of Warsaw (consent no KB/93/2015, dated 5 May 2015). A pilot study including 20 women was conducted before the study; the questionnaire was modified to obtain the Cronbach’s alpha coefficient of 0.81. Properly and fully completed (up to 3 years after childbirth) questionnaires were included in the analysis.

The obtained data were analysed statistically using the Chi-square test and correlation analysis using the Spearman rank correlation coefficient. The strength of the relationship between two variables was assessed using J Guilford’s classification scale: for r >0.0 – ≤0.1 as slight; r = >0.1 – ≤ 0.3 as low; r= >0.3 – ≤ 0.5 as moderate; r = >0.5 – ≤0.7 as high; r = >0.7 – ≤0.9 as very high; r = >0.9 – <1.0 as almost complete; r = 1 as complete. Furthermore, odds ratio (OR) along with 95% confidence interval, including unadjusted OR and adjustment for sociodemographic variables were determined for selected categorised variables based on logistic regression. Statistica 12 (Statsoft) was used for statistical analysis. A statistical significance level of 0.05 was used.

## RESULTS

A total of 2480 out of 3455 completed questionnaires were included in the analysis (16 questionnaires were completed incorrectly, 959 were completed at a later time than 3 years after childbirth). The questionnaires were completed after an average of 1.15 ± 0.81 years. The age at childbirth ranged from 13 up to 43 years (mean age 27.24 ± 3.98 years); the age at the time of questionnaire ranged from 13.1 to 45.4 years (mean age 28.38 ± 4.00 years). A total of 1757 (70.8%) respondents were primagravidas. The sociodemographic data of respondents as well as data on the course of pregnancy, answers to questions regarding oral health and basic health behaviours during pregnancy are presented in the tables (Tables 1 and 2). Correct answers to all questions regarding oral health were given by 515 (20.8%) women.

**Table 1:** Sociodemographic characteristics and the course of pregnancy of respondents

Parameters	N (%) 2480 (100)
**Age at delivery (years)**	
≤20	110 (4.4)
21–30	1889 (76.2)
>30	481 (19.4)
**Place of residence**	
rural area	656 (26.5)
small town	786 (31.7)
big city	1038 (41.9)
**Education**	
primary/middle/basic vocational	72 (2.9)
secondary	504 (20.3)
incomplete higher/higher	1904 (76.8)
**Occupational activity/during education**	1967 (79.3)
**Financial status**	
bad	217 (8.8)
average	1231 (49.6)
good or very good	1032 (41.6)
**Regular attendance to dental office before pregnancy**	1248 (50.3)
**General medical problems during pregnancy**	776 (31.3)
risk of premature birth	385 (15.5)
diabetes	202 (8.1)
hypertension	214 (8.6)
preeclampsia	41 (1.6)
hyperthyroidism	53 (2.1)
hypothyroidism	475 (19.2)
gestational cholestasis	44 (1.8)
**Delivery**	
vaginal	1527 (61.6)
Caesarean section (planned)	411 (16.6)
Caesarean section (emergency)	542 (21.9)
before the 37th gestational week	162 (6.5)


**Table 2:** Oral health knowledge among respondents and their health behaviours during pregnancy

	Oral health knowledge among respondents		N (%)
1.	Are dental check-ups in pregnancy important for oral health?	yes	2309 (93.1)
		no	54 (2.2)
		not known	117 (4.7)
2.	Does fluoride prophylaxis influence dental condition in pregnancy?	yes	2169 (87.5)
		no	57 (2.3)
		not known	254 (10.2)
3.	Do changes of the diet influence oral health in pregnancy?	yes	1647 (66.4)
		no	306 (12.3)
		not known	527 (21.3)
4.	Does presence of caries in the mother favour caries in the child?	yes	1766 (71.2)
		no	130 (5.2)
		not known	584 (23.5)
5.	Is dental treatment safe in pregnancy?	yes	1961 (79.1)
		no	90 (3.6)
		not known	429 (17.3)
**Health behaviours in pregnancy**			
1.	Toothbrushing	rarely	46 (1.8)
		once a day	384 (15.5)
		at least twice a day	2050 (82.7)
2.	Use of fluoride toothpaste	yes	1979 (79.8)
		no	164 (6.6)
		not known	337 (13.6)
3.	Cigarette smoking	passive	243 (9.8)
		active	69 (2.8)
4.	Dental office attendance (total)		1594 (64.3)
	need of prophylactic treatment		505 (20.4)
	referral from a gynaecologist		476 (19.2)

A statistically significant correlation was found between the level of knowledge about oral health and health behaviours of respondents and sociodemographic factors (Table 3). No statistically significant correlations were observed between the level of dental knowledge and parameters describing the general condition of a pregnant woman or the mode of delivery. Also, there was no statistically significant correlation between pregnancy/childbirth parameters and health behaviours. Only the relationship between dental appointment due to a referral from the gynaecologist and general medical problems during pregnancy (r = 0.052) and the risk of premature birth (r = 0.053) was statistically significant.

**Table 3:** **Table 3** Spearman’s correlation coefficients indicating factors influencing women’s oral health behaviour during pregnancy

Parameters	Correct answers to all questions regarding oral health	Toothbrushing		Smoking		Visit at dental office during pregnancy		Proper oral hygiene and dental care in pregnancy, no exposition to cigarette smoke
		at least twice a day	fluoride containing toothpaste	passive	active	total	prophylactic treatment	
Correct answers to all questions regarding oral health	–	0.235*	0.258*	–0.089*	–0.063*	0.158*	0.109*	0.155*
Age at childbirth	0.148*	0.169*	0.188*	–0.161*	–0.130*	0.089*	0.117*	0.144*
Age at questionnaire completion	0.147*	0.159*	0.208*	–0.167*	–0.130*	0.088*	0.108*	0.138*
Place of residence (big city)	0.088*	0.120*	0.082*	–0.045*	0.009	0.060*	0.055*	0.058*
High level of education	0.132*	0.177*	0.124*	–0.188*	–0.206*	0.092*	0.051*	0.081*
Occupational activity	0.063*	0.077*	0.058*	–0.059*	–0.083*	0.070*	0.035	0.049*
Financial situation (good/very good)	0.116*	0.133*	0.052*	–0.108*	–0.073*	0.057*	0.098*	0.110*
Financial situation (bad)	–0.082*	–0.088*	–0.043*	0.085*	0.060*	–0.052*	–0.039*	–0.058*
Risk of preterm birth	–0.052*	–0.011	0.015	0.043*	0.016	0.027	–0.008	–0.008
Regular attendance to dental office before pregnancy	0.510*	0.145*	0.097*	–0.088*	–0.062*	0.240*	0.159*	0.187*

* statistical significance.

In the simple logistic regression analysis assessing the impact of the strongest variable – ‘regular dental visits in the period preceding pregnancy’ on the knowledge of correct answers to all questions OR reached the value of 429.2 (CI:106.7–1726.2; p <0.001). The OR was 1109.6; (CI:156.8–7852.2; p <0.001) in the model including sociodemographic variables.

The highest correlation coefficients were observed between hygienic behaviours and the level of knowledge on oral health, as well as between dental visits during pregnancy and the regular use of dental care before pregnancy. Correct answers to all questions increased the chance of brushing the teeth at least twice during a day (unadjusted OR = 289.1; CI:18.02–4637.3; p <0.001, adjustment OR = 159.2; CI:22.5–1127.5; p <0.001), the use of fluoride toothpaste (unadjusted OR = 353.1; CI: 22.0–662.8; p <0.001, adjustment OR = 196.3; CI: 27.7–1390.0; p <0.001), dental appointment during pregnancy (unadjusted OR = 1.66; CI:1.35–2.04; p <0.001, adjustment OR = 1.41; CI:1.03–1.92; p = 0.035), and a simultaneous implementation of all the above-mentioned practices (unadjusted OR = 2.44; CI:1.93–3.07; p <0.001; adjustment OR = OR=1.72; CI:1.39–2.14; p <0.001). Correct answers to all questions reduced the risk of passive and active smoking during pregnancy (unadjusted OR = 2.79; CI:1.83–4.26; p <0.001, adjustment OR = 2.11; CI:1.50–2.96; p <0.001). Similarly, regular dental visits during the prepregnancy period increased the chance of proper hygiene, visiting the dentist during pregnancy and non-smoking (unadjusted OR = 2.88; CI: 2.29–3.63; p <0.001, adjustment OR =1.82; CI: 1.47–2.26; p <0.001), as well as continued dental care throughout pregnancy (unadjusted OR = 2.79; CI: 2.35–3.32; p <0.001, adjustment OR = 2.05; CI:1.47–2.86; p <0.001). Conviction that the visit is safe was another factor associated with reporting for dental appointments during pregnancy (r = 0.195). The OR was 2.47 (CI: 2.09–2.93; p <0.001) for the model taking into account the impact of the conviction about dental visit safety on attending dental appointment. This factor was, however, insignificant in a model considering other sociodemographic factors (OR = 1.24; CI: 0.84–1.84; p = 0.285).

## DISCUSSION

Health-related practices among pregnant women, such as avoiding stimulants, proper eating habits and hygiene practices, as well as regular dental check-ups are essential for maintaining oral health. Despite some limitations of our study, in which data were collected through an electronic self-administered questionnaire (inclusion of women with access to the internet, who have the ability to use electronic questionnaires and wish to present themselves in a good light), we revealed some deficiencies in the basic health knowledge and practices among pregnant women. The importance of caries prevention in pregnancy was not understood by every tenth, and the importance of proper dietary habits was not understood by every fourth woman. A larger number of women, however, knew about the relationship between the oral health of the mother and the child. Similar results were obtained by other researchers. In Australia and Canada, about half of the women surveyed knew that there was a relationship between oral health in the mother and the health of her child.^3,22^ In our study, not all respondents cleaned their teeth at least twice a day and not all of them used toothpaste with fluoride.

In Australia, the percentage of women who clean their teeth at least twice a day ranges from 56.2% to 70.4%.^22,29^ A similar proportion (73.7%) was reported in a group of immigrants in North London.^27^ In the USA, 83% of pregnant women reported brushing their teeth at least once or twice a day.^7^ Significantly better health behaviours during pregnancy were reported for women in Denmark, where 96% of 1935 pregnant women brushed their teeth at least twice a day at the turn of 1998 and 1999.^12^ Most Australian women (94.7–98.3%) used fluoride toothpaste.^22,29^ In our study, 6.6% of respondents declared that they used fluoride-free toothpaste, and 12.7% were unsure whether their toothpaste contained fluoride. In Canadian studies, up to 15.9% of women did not use fluoride toothpaste, and 11.6% of respondents had no knowledge of the fluoride content in their toothpaste.^3^ This may be due to a misbelief about negative systemic effects of fluoride exposure. Brazilian researchers have noted that some women (8.3%) generally discontinue using toothpaste during pregnancy due to nausea. They also observed reduced frequency of daily toothbrushing.^53^ Similarly, in Canadian studies during pregnancy, 19.2% of mothers reported difficulties with brushing.^3^

George et al showed in their studies that although 97.9% of pregnant women understood the importance of regular dental visits, more than a third did not attend such a visit during pregnancy.^22^ Similarly, although 93.1% of our respondents were aware of the importance of dental check-ups for oral health, only 64.3% reported this visit during pregnancy.

The obtained results indicate some positive changes in pregnancy-related attitudes among both women and medical personnel over the last 13 years. A questionnaire including 1380 pregnant women, which was conducted in 2006 as part of the National Monitoring of Oral Health and Its Determinants funded by the Ministry of Health, showed that less women (53%) visited a dentist during pregnancy and only 3% were referred by a gynaecologist.^47^

Health behaviours are shaped by many factors that include individual factors (ie, upbringing, personality, sociodemographic factors) as well as environmental factors (society, media, culture). Similarly to other authors, we have shown the influence of sociodemographic factors on the level of dental knowledge and health behaviours of pregnant women despite the fact that the population we analysed, unlike populations studied in other countries, was culturally and ethnically homogenous.^3,7,22,29^ Positive health attitudes among women were influenced by living in an urban area, professional work or continuing education, good financial situation and high level of education. In our study the level of knowledge and oral practices improved with age. However, sociodemographic factors had a greater impact on oral hygiene behaviours rather than a dental visit during pregnancy, which may to a certain extent result from the availability of free dental care in Poland. Malaysian studies did not show any correlation between dental visit and maternal age, ethnic group, level of education, household income or employment status.^42^

Dental attendance during pregnancy varies in different world regions, ranging between 12.6% and 88.8%.^3,4,7,8,^^29,35,41,42,45,47^ There are also different barriers to accessing dental care by pregnant women. These include, among other things, costs.^3,22,29^ Keirse and Plutzer showed that costs were reported as barriers by 26.9% and the lack of private dental care insurance by 82.4% of women.^29^ However, these factors should not be of great importance in countries where dental treatment is free for women during pregnancy. On the other hand, dental attendance is insufficient even in countries providing free dental care to pregnant women.^18,27,31,34,41,42,47^ Studies among immigrant women in North London have shown that over one-third of respondents were not aware of the availability of free dental care during pregnancy, with only one-third of them visiting a dentist.^27^ In Polish population, except for tooth extraction and endodontic treatment, all prophylactic and treatment procedures during pregnancy were usually performed in private practices rather than those having a contract with the Polish National Health Fund.^31^

In our study population, regular use of dental care before pregnancy and conviction about the safety of dental visit during pregnancy were the two main factors increasing the chance of dental appointment. Similar observations were made by other authors. Boggess et al showed that the lack of routine dental care before pregnancy was the most common predictive factor for the lack of dental care during pregnancy (OR = 4.35 (2.5–7.69)).^7^ Furthermore, ethnic factors, age over 36 years, annual income below USD 30,000, education lower than secondary and lack of private insurance also had negative effects.^7^ In contrast to the studies cited, we observed that age had a positive influence in the case of Polish women. The importance of educational level, dental insurance and income level was also noted by Canadian researchers.^7^ Positive effects of the level of education on health attitudes among pregnant women is also recognised by many researchers.^32^ However, studies in Greece showed that women with higher education were less likely to visit a dentist.^18^ According to the authors, this may result from professional activity, which makes these women more occupied.^18^ Similarly, Amin et al showed that 22% of women who did not attend a dentist claimed that they were too busy to attend such an appointment.^3^ On the other hand, Saddki et al showed that women claimed that they did not attend dental appointments since they were busy with household chores (30.7%) or busy at work (38.6%).^42^ Our study showed that both high education level and occupational activity/studying during pregnancy had positive effects on women’s health attitudes. Bamanikar and Kee found that better educated and professionally active women were more knowledgeable about dental treatment.^4^ The authors concluded that the more extensive knowledge was probably due to social communication. However, they showed no relationship between the level of knowledge and dental visits during pregnancy. In Canada, alike in Poland, mothers with more knowledge about possible connections between oral health and pregnancy and those who regularly visited dental office before pregnancy were more likely to visit the dentist during pregnancy.^3^ The authors concluded that a perceived need, habit of regular dental visits, and access to dental services are main predictors for the use of dental care during pregnancy.^3^

In our study, the level of oral health knowledge among respondents was the strongest predictor of health practices during pregnancy. Deficiencies in the basic knowledge on oral health were associated with more than a twofold increase in the risk of inappropriate behaviour during pregnancy (ie, hygiene negligence, failure to report to the dentist and passive or active smoking). Saddki et al found a statistically significant association between maternal visit to dental clinic and oral health education received before the current pregnancy and an awareness of the association between poor maternal oral health and adverse pregnancy outcomes, with OR of 5.03 (95% CI: 2.15–11.76) and 4.57 (95% CI: 1.73–12.06), respectively.^42^ The increased risk of avoiding dental visits during pregnancy could be due to anxiety associated with a visit to the dental office.^7,8^ In our study population, almost one in five respondents was uncertain about the safety of dental visit during pregnancy or considered such a visit to be unsafe. Boggess et al showed that 12.6% of respondents agreed that ‘it is not safe for pregnant women to get routine dental care such as check-ups and cleanings’, whereas 36.6% were uncertain.^8^ Dental anxiety is a well-known factor limiting the use of dental care. As shown by Keirse and Plutzer, 11.7% of pregnant women reported that they often postponed appointments due to dental fear.^29^

The harmful effects of smoking tobacco on the health of the woman and her child is beyond doubt.^26,33,43^ In our study population, exposure to tobacco smoke during pregnancy was reported by one in ten women. Active smoking was reported by 2.8% of respondents. Cross-sectional studies conducted in Canada and Europe showed that the percentage of pregnant smokers was many times higher than in Poland. A total of 23% of Canadians, 22.3% of Italians and 27.7% of Germans smoked during pregnancy.^14,20,44^ Our results were also lower compared to a nationwide Polish study conducted in 2012 in a group of 2758 women, which showed that 7% of respondents smoked tobacco.^50^ Perhaps the low percentage of pregnant smokers in Poland, which dropped within a few years, is due to antismoking campaigns conducted in the country. However, this should be verified in future studies.

It would seem that the very fact of becoming pregnant should be one of the factors influencing health behaviours in pregnancy. Pregnancy causes most women to increase their interest in matters of their own health. Gupta et al, who assessed oral health knowledge, practices and attitude of pregnant and non-pregnant women, showed that a change in the attitude towards dental care, which increases the use of dental care in the future, occurs during pregnancy.^24^ Our analysis did not confirm the statistically significant role of most factors related to the course of pregnancy or mode of delivery in health behaviours during pregnancy. However, there were some relationships between dental visits of women referred by their gynaecologists and general health issues as well as the risk of preterm birth. It is worth noting that we have also found a negative correlation between the conviction about the safety of dental visits and the risk of premature birth. Furthermore, a relationship between women’s knowledge on the need for dental check-ups and elective C-sections was observed. This indicates a greater involvement of gynaecologists in the education and motivation of women for oral healthcare in the case of complicated pregnancy. It also confirms the authority of a gynaecologist among pregnant women. This was observed in a study of 3455 Polish women in which dental appointments were upheld by 87.3% of women referred by a gynaecologist and by 56.9% of those without a referral (OR = 5.20 (4.05–6.67); p <0.001).^30^ Among those who were referred, dental appointments were upheld in 91.7% of cases when further asked to provide oral health feedback and in 83.5% of cases in absence of such further request (OR = 2.19 (1.3–3.66); p = 0.003).^30^

## CONCLUSION

Our study showed that health attitudes related to oral health during pregnancy are modified by sociodemographic factors. The obtained results point to the need for educating women from rural areas, young women, women with low level of education and/or low socioeconomic status, as well as those non-working/non-studying. High level of oral health knowledge and the use of dental care before pregnancy are the main predictors for appropriate health practices. Dental attendance among pregnant women is additionally influenced by the awareness of the safety of dental visits and a referral from a gynaecologist in case of general condition and risk of premature birth.
